# A Potential Therapeutic Effect of Suxiao Jiuxin Pills in Treating Postacute Sequelae of COVID-19: Case Report

**DOI:** 10.1155/crdi/4713552

**Published:** 2024-11-21

**Authors:** Li Sun, Nannan Li, Huilin Li, Qingshan Zhang, Shuai Bao, Xiaolu Li

**Affiliations:** Department of Emergency Medicine, The First Affiliated Hospital of Shandong First Medical University & Shandong Provincial Qianfoshan Hospital, Jinan, Shandong 250014, China

**Keywords:** long COVID, PASC-CVS, Suxiao Jiuxin Pills

## Abstract

Postacute Sequelae of COVID-19 Cardiovascular Syndrome (PASC-CVS) refers to a broad spectrum of cardiovascular symptoms that manifest four weeks or more after infection with COVID-19, which cannot be diagnosed as cardiovascular disease through standard examinations. Common symptoms include exercise intolerance and tachycardia, alongside persistent issues such as chest pain, chest tightness, and difficulty breathing. PASC-CVS significantly affects patients' quality of life; however, effective treatments for this condition are currently lacking. In this report, we present two cases of PASC-CVS patients who experienced well-controlled cardiovascular symptoms following treatment with Suxiao Jiuxin Pills. Our findings may offer a novel approach to the clinical management of PASC-CVS.

## 1. Introduction

The severe impact of COVID-19 on global healthcare represents one of the most significant public health challenges in recent decades. COVID-19 affects not only the respiratory system but also various other organs and systems, resulting in a diverse array of local and systemic clinical manifestations [1]. Long COVID-19 is defined as a condition that occurs in individuals with a confirmed or suspected history of COVID-19 infection, typically manifesting within 3 months of the onset of the initial illness. This condition is characterized by symptoms and effects that persist for at least 2 months and cannot be explained by other medical diagnoses [2]. Symptoms of long COVID-19 encompass a wide range of cardiopulmonary and neurological issues, including persistent shortness of breath, chest pain, fatigue, headaches, cognitive impairment often referred to as “brain fog,” and palpitations [3]. Among them, Postacute Sequelae of COVID-19 Cardiovascular Syndrome (PASC-CVS) is characterized by a variety of cardiovascular symptoms that emerge 4 weeks or more after SARS-CoV-2 infection, with no evidence of cardiovascular disease (CVD) based on standard examinations [4, 5].

A survey involving a cohort of 3700 self-reported patients with PASC from 56 countries revealed that, although only 8% of participants were hospitalized, 90% experienced symptoms lasting more than 35 weeks. Among these patients, half were unable to return to work after 6 months, with 83% reporting symptoms such as chest pain, palpitations, and tachycardia [6]. The pathogenesis of PASC-CVS remains incompletely understood. Potential contributing factors include inflammation and immune activation, the persistent presence of the virus and reactivation of latent viruses, endothelial dysfunction, exercise-related metabolic injury, and decreased cardiac adaptability resulting from SARS-CoV-2 infection [7]. Furthermore, the absence of CVD in routine examinations has contributed to a lack of effective treatments for PASC-CVS.

Suxiao Jiuxin Pills are a well-known contemporary Chinese medicine formula primarily composed of Chuanxiong and borneol. These pills are recognized for their ability to promote qi and blood circulation, eliminate stasis, and relieve pain. As a first-line treatment for angina pectoris associated with coronary heart disease, Suxiao Jiuxin Pills act quickly and demonstrate significant therapeutic effects without evident toxic side effects. The multicomponent nature of these Chinese herbal ingredients allows Suxiao Jiuxin Pills to enhance myocardial oxygen supply, alleviate coronary spasms, improve microcirculation, and support endothelial function. Additionally, they exhibit anti-inflammatory, analgesic, and sedative properties [8–13]. Many Chinese individuals report using these pills to alleviate cardiac-related discomforts, noting improvements in PASC-CVS following their use. In this study, we examined the effects of Suxiao Jiuxin Pills on patients with PASC-CVS, presenting two representative cases. Our findings indicate that, in patients with limited therapeutic response to other medications, cardiovascular symptoms were effectively managed following treatment with Suxiao Jiuxin Pills. This suggests that Suxiao Jiuxin Pills may be a promising therapeutic option for patients suffering from PASC-CVS.

## 2. Case Report

### 2.1. Case 1

A 37-year-old man was admitted to the Department of Emergency Medicine, the First Affiliated Hospital of Shandong First Medical University due to persistent discomfort in the precordial area lasting over five months, which had worsened in the preceding day. The patient reported that the episodic discomfort began after contracting SARS-CoV-2 more than 5 months prior. During these episodes, he experienced palpitations without significant chest pain and reported no noticeable decline in exercise tolerance. Despite undergoing multiple electrocardiograms and myocardial enzyme tests during his visits to the hospital, no abnormalities were detected. The patient intermittently used oral medications, including Betaloc, coenzyme Q10, fluoxetine, and Meriquin tablets; however, these treatments yielded poor therapeutic effects and negatively impacted his quality of life. Notably, the patient had a history of good health with no prior incidence of coronary heart disease.

**Figure 1:**
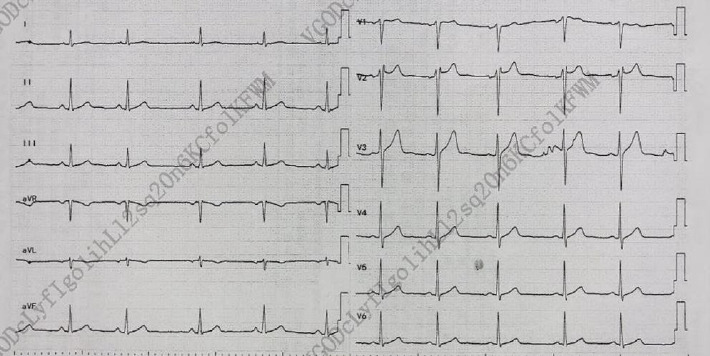
Sinus arrhythmia and no significant ischemic changes as suggested by the electrocardiogram for Case 1.

**Figure 2:**
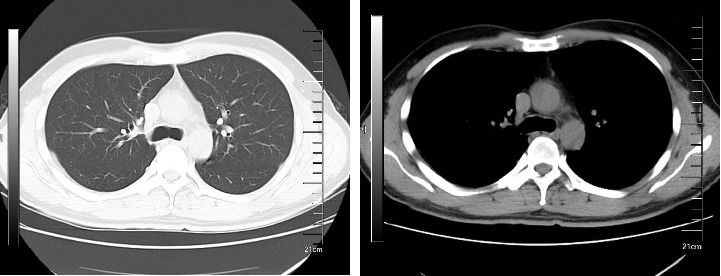
Bilateral lung nodules as indicated by the chest CT, and recommended follow-up for Case 1.

**Figure 3:**
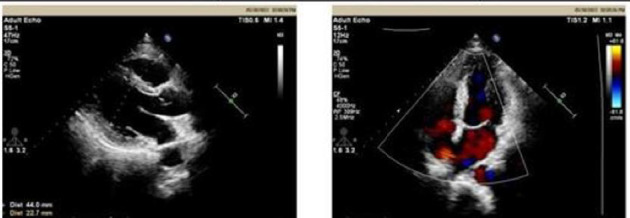
Cardiac ultrasound reveals no significant abnormalities detected in cardiac structure and blood flow for Case 1.

**Figure 4:**
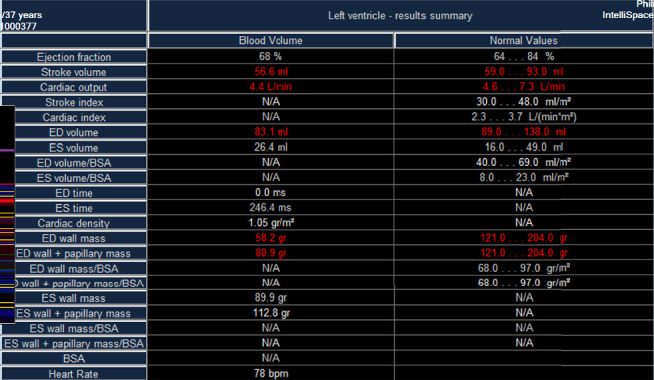
No obvious abnormal changes such as myocardial edema as indicated by the cardiac magnetic resonance imaging for Case 1.

Upon admission, myocardial enzyme tests revealed no abnormalities. The electrocardiogram indicated sinus arrhythmia (Figure 1), while cardiac ultrasound and chest CT scans showed no significant abnormalities (Figure 2). The dynamic electrocardiogram did not reveal any evidence of myocardial ischemia or arrhythmia. Furthermore, the echocardiogram demonstrated no significant structural or hemodynamic abnormalities (Figure 3), and cardiac magnetic resonance imaging also showed no irregularities (Figure 4). The patient exhibited widespread cardiovascular symptoms following a COVID-19 infection. Standard diagnostic tests, including myocardial perfusion studies, did not provide objective evidence of CVD. Consequently, a diagnosis of PASC-CVS was established. Prior to admission, the patient had been taking various medications aimed at myocardial nourishment and ventricular rate control. Upon admission, the treatment regimen was supplemented with 8 capsules of Suxiao Jiuxin Pills, administered three times daily. Notably, 2 days postadmission, the patient's symptoms significantly improved, leading to his discharge from the hospital. During a 1-month follow-up, the patient reported no further discomfort in the precordial area.

### 2.2. Case 2

**Figure 5:**
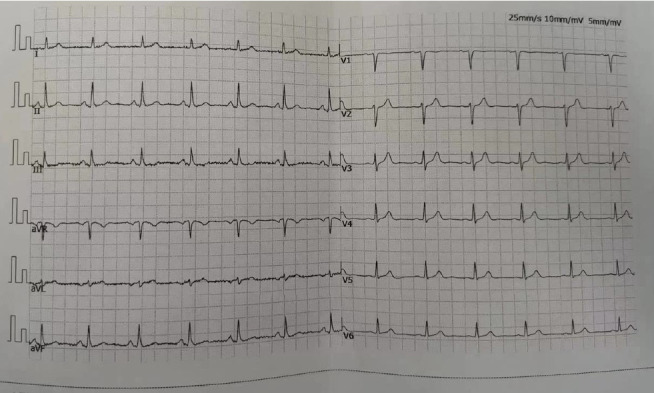
The electrocardiogram (ECG) examination indicates sinus rhythm with a generally normal ECG pattern for Case 2.

**Figure 6:**
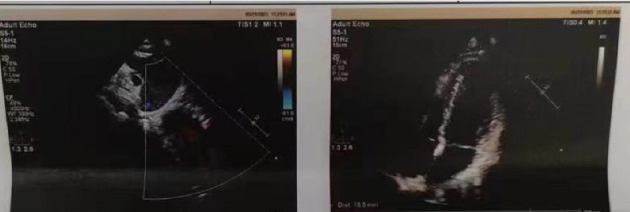
Cardiac ultrasound: no significant abnormalities detected in cardiac structure and blood flow for Case 2.

**Figure 7:**
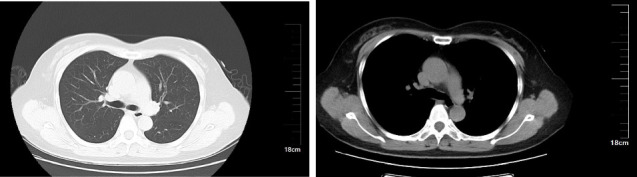
No significant abnormalities observed in chest CT for Case 2.

**Figure 8:**
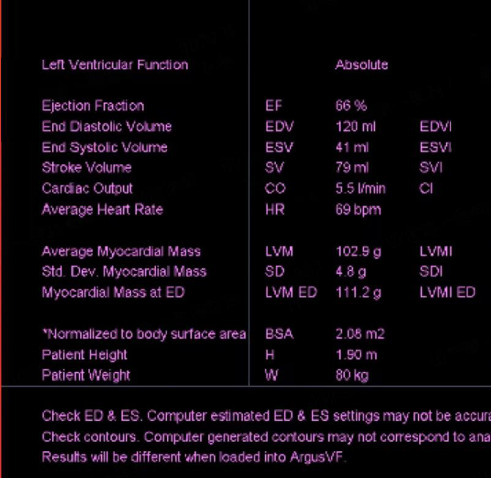
Myocardial perfusion: no definitive abnormalities observed on non-contrast and contrast-enhanced cardiac imaging for Case 2.

A 34-year-old man was admitted to the Department of Emergency Medicine, the First Affiliated Hospital of Shandong First Medical University due to persistent chest tightness that had lasted for 3 months. The patient reported experiencing chest tightness and mild dyspnea, which were not related to physical activity, following a SARS-CoV-2 infection 3 months prior. Despite multiple hospital visits, he had not found effective relief from his symptoms. Upon admission, myocardial enzyme tests were performed and revealed no abnormalities. Additionally, a series of examinations, including an electrocardiogram (Figure 5), cardiac ultrasound (Figure 6), chest CT (Figure 7), and cardiac MRI (Figure 8), failed to provide definitive evidence of CVD. Based on the World Health Organization's definition, a diagnosis of PASC-CVS was also considered. The patient was prescribed an outpatient treatment regimen consisting of 8 capsules of Suxiao Jiuxin Pills, to be taken three times daily after meals. During follow-up, the symptom of chest tightness was significantly controlled.

## 3. Discussion

According to the expert consensus of the American College of Cardiology (ACC), cardiovascular symptoms associated with PASC are categorized into two types: PASC-CVD and PASC-CVS. Patients with PASC-CVD develop cardiovascular symptoms with identifiable causes more than 4 weeks after SARS-CoV-2 infection. These symptoms primarily include myocarditis or myocardial injury, pericarditis, new or exacerbated myocardial ischemia due to coronary obstruction, nonischemic cardiomyopathy, pulmonary hypertension, right heart failure, and arrhythmias [14]. In contrast, the most common symptoms observed in patients with PASC-CVS include reduced exercise tolerance, tachycardia, chest pain, and dyspnea; however, no evidence of cardiovascular lesions can be detected in these cases [4]. Diagnostic evaluation typically involves basic laboratory tests, including CBC, BMP, CTn, CRP, ECG, echocardiogram, ambulatory electrocardiogram, chest X-ray or CT, and cardiac magnetic resonance imaging [15]. Coronary computed tomography angiography (CTA) may also be utilized if necessary. The two cases reported above exhibited long-term cardiovascular symptoms following SARS-CoV-2 infection, with no evidence of CVD, thereby supporting the diagnosis of PASC-CVS.

Although the pathogenesis of PASC-CVS is not yet fully understood, several mechanisms may underlie its primary symptoms: (1) Reduced exercise tolerance and tachycardia may arise from SARS-CoV-2-induced alterations in immune activity and metabolic functions, leading to manifestations similar to chronic fatigue syndrome. (2) Isolation. Decreased physical activity, and increased bed rest can contribute to diminished cardiovascular adaptability. This deconditioning may result in decreased blood volume, reduced venous return, myocardial atrophy, and decreased cardiac output per beat. Additionally, it may lead to diminished vagal tone and heightened sympathetic nerve excitability, ultimately resulting in tachycardia and/or orthostatic hypotension, akin to postural orthostatic tachycardia syndrome. (3) Chest pain. The effects of SARS-CoV-2 and the subsequent immune response can cause damage to vascular endothelial cells, which may impair coronary endothelial function and precipitate coronary spasms and myocardial ischemia [7]. A study found that 83% of PASC patients with suspected angina pectoris exhibited abnormal coronary endothelial function [16]. Furthermore, latent pericardial and myocardial diseases may also contribute to chest pain [17].

Thus far, effective treatments for PASC-CVS remain elusive due to unclear underlying mechanisms. After ruling out serious CVD, most patients should be advised to follow these guidelines: (1) Implement appropriate exercise regimens to increase myocardial mass and blood volume, improve myocardial compliance, and enhance both stroke volume and maximum oxygen uptake. (2) Consider prescribing medications such as beta-receptor antagonists and calcium channel blockers, as well as ivabradine, to manage tachycardia. In cases where endothelial dysfunction is suspected, calcium antagonists or long-acting nitrates may be beneficial. For persistent chest pain resulting from microvascular functional abnormalities, nutritional therapies, such as beetroot extract and L-arginine can be explored to elevate nitric oxide levels and improve microvascular function. (3) We recommend that patients receive psychological support if therapeutic interventions yield suboptimal results.

Despite the various treatments administered, the outcomes have not been satisfactory. For instance, in the two cases we report, the patients repeatedly visited the hospital due to cardiovascular symptoms, yet they did not experience significant improvement despite undergoing several drug therapies, including mood-regulating medications. PASC with cardiovascular manifestations poses considerable challenges to the patients' daily lives and professional activities, highlighting the urgent need for more effective treatment options and pharmacological interventions.

In the two cases reported above, the application of Suxiao Jiuxin Pills has demonstrated significant therapeutic effects. These patients are representative of typical cases encountered in our clinical practice. We hypothesize that the underlying mechanisms may be associated with the primary active components of Suxiao Jiuxin Pills, specifically Chuanxiong and borneol. Analyses utilizing UPLC/Q-TOF-MS and a dual-fluorescence screening system have identified that the principal components of Suxiao Jiuxin Pills include phenanthrene derivatives (notably butylphenanthrene, gaozaban lactone, and foreign Chuanxiong lactone), alkaloids (predominantly Chuanxiong), phenolic acid compounds (such as ferulic acid), and borneol-derived camphor.

Chuanxiong, foreign Chuanxiong lactone, and ferulic acid have been found to exhibit notable calcium antagonistic effects, which can effectively alleviate myocardial ischemia and coronary spasm. As a classic traditional Chinese medicine, Chuanxiong is known for its positive impact on microcirculation. Additionally, camphor, recognized as a traditional “guiding” drug, can rapidly cross the blood-brain barrier, leading to a reduction in heart rate, improved oxygen consumption, decreased levels of inflammatory factors (including TNF-*α* and IL-6), and mitigation of inflammatory responses. It also regulates nitric oxide levels and demonstrates analgesic and sedative properties. Collectively, these effects may significantly enhance the symptoms of patients with PASC-CVS, highlighting the multitarget treatment advantages inherent in traditional Chinese medicine [8–13]. Suxiao Jiuxin Pills may prove effective for patients with PASC-CVS. Importantly, this formulation consists entirely of Chinese herbal ingredients and is associated with fewer adverse reactions. The most frequently reported side effects include gastrointestinal irritations such as nausea, vomiting, and gastric discomfort. In cases where oral administration is intolerable, sublingual administration may be considered. However, it is important to note that this medication is contraindicated in pregnant women [8–13].

PASC-CVS currently lacks effective treatment options. The two cases presented here provide a new approach and effective strategy for treating patients with PASC-CVS. In future clinical practice, we may consider utilizing Suxiao Jiuxin Pills to alleviate symptoms in PASC-CVS patients and improve their quality of life. Certainly, for different patients, it is important to provide corresponding symptomatic treatment based on their distinct clinical manifestations. For example, administering *β*-blockers for patients with tachycardia and providing treatment to improve sleep for those with severe sleep disturbances. This also offers insights into the potential advantages of traditional Chinese medicine, which often operates through multitarget mechanisms, particularly in situations where other treatments have proven ineffective. It is important to emphasize that the application of Suxiao Jiuxin Pills for the treatment of PASC-CVS requires additional clinical evidence and the support of randomized controlled trials. Currently, we are conducting a multicenter observational clinical study aimed at providing further evidence in this area.

## 4. Conclusion

Suxiao Jiuxin Pills exhibited effective management of cardiovascular symptoms for patients with PASC-CVS and limited responses to other treatments. These observations indicate the potential of Suxiao Jiuxin Pills as a promising therapeutic option for patients suffering from PASC-CVS.

## Data Availability

The data generated and/or analyzed during the current study can be requested from the corresponding author upon reasonable request.
